# Letter to the editor, concerning the article by Latzkow, Schmit et al. about orthodontic materials in MRI

**DOI:** 10.1007/s00784-025-06537-5

**Published:** 2025-09-15

**Authors:** Felix H. Blankenstein

**Affiliations:** Department of Prosthodontics, Geriatric Dentistry and Craniomandibular Disorders, Charité – Universitätsmedizin Berlin, Aßmannshauser Str. 4-6, 14197 Berlin, Germany

Dear Editor,

The study ‘Orthodontic appliances and their diagnostic impact to brain MRI’ (doi.org/10.1007/s00784-025-06275-8) recently published by Latzkow and Schmit et al. in Clinical Oral Investigations examines an important topic, but has methodological deficits that substantially limit its clinical significance.

As early as 1996, Schenck [1] described in detail the decisive role of the magnetic susceptibility of metallic objects in MRI. If this fundamental work had received more attention in medical circles, we would have been spared from numerous methodologically inadequate publications on this topic. Unfortunately, the current study also makes two of these typical mistakes:

1. Individual metal objects are considered representative of an entire alloy group. In the current study, this refers to three different types of stainless steel from which the analysed metal objects are made: the brackets, the molar bands and the transpalatal arch.
2. The products analysed are not specified in a comprehensible manner. The authors name the manufacturer, but not the material classification. The general term “stainless steel” is not sufficient, as the group of approx. 120 stainless steel types is also very heterogeneous as regards to their magnetizability.

Our research revealed that the brackets used in the first experiment of the current study were made from nickel-free austenitic stainless steel X15 CrMnMoN 17 11 3. In order to achieve a stable austenitic structure despite the absence of nickel, this steel was nitrided, giving it an almost nonmagnetic quality.

The stainless steel molar bands used in the second experiment were made from a stable austenite alloy with 12% nickel (DIN/EN 1.4303, equivalent to X4 CrNi 18 12), which is also non-magnetic.

The transpalatal arch in the third experiment consists of a metastable austenite (DIN/EN 1.4310) which, as a result of cold deformation (e.g. bending, upsetting, drawing), undergoes a martensitic transformation and thus becomes magnetizable. This is exactly what happens when the orthodontist adapts the arch, which is supplied flat by the manufacturer, to the individual shape of the palate. The results of this study were therefore predictable from a materials science point of view: the stable austenites caused only minor local artifacts. However, the martensitically transformed palatal arch produces such large artifacts in sensitive MRI sequences that over 80% of the brain regions evaluated are diagnostically unusable. This study would therefore be only helpful for radiologists and orthodontists if their patients wore exactly the here examined products in their mouths. Given the variety of orthodontic metal products on the market, this should only rarely occur. Even after this study, the fundamental problem remains: there is a lack of material data on the magnetizability of orthodontic products. The reason for this is an exemption in the European Medical Devices Regulation: the magnetizability does not have to be specified for metal products for intraoral use. And the majority of manufacturers rely on this exemption. To provide clarity about expected artifacts prior to an MRI, there are currently only two non-destructive methods.

1. Many radiologists utilize a simple “in-house” magnet test: If a bar magnet sticks to the respective products in question, it is assumed that they are magnetizable and will cause artifacts. However, there is no valid methodology for this (yet). This is because the permanent magnets used are not defined in terms of size, mass, field strength, field configuration, and the ratio of mass to field strength. Furthermore, this test cannot be used to estimate the degree of magnetizability.
2. The relative magnetic permeability can be measured intraorally. This would require a specially modified measuring device, for which we have already published a proposal. [2]

Nevertheless, I believe that this study by Latzkow, Schmit et al. offers great potential thanks to its sophisticated radiological methodology in terms of planning, implementation, and evaluation. After researching the relevant material data, this study design offers a great opportunity to determine the formation of artifacts caused by metal products with varying degrees of magnetizability in the different sequences and main field strengths, either metrically or anatomically. Perhaps this could lead to an “Innsbruck list of MR compatibility of orthodontic products”? Or an internationally recognized “Innsbruck hallmark of approval for artifact-free dental metal products” could be awarded? In the best-case scenario, a series of studies supplemented by the material data could even gradually lead to a market shakeout if products that generate strong artifacts are labeled accordingly.

But dont get me wrong: I dont want to stigmatize magnetizable products! If certain functions can only be achieved through certain material properties, then magnetizability may have to be accepted. If an MRI is indicated for a wearer of such products, Radiologists can then decide on the basis of valid data whether the product can remain in situ or must be removed.

With collegial regards to the working group led by Lisa Latzkow and Anna Schmit.

Felix H. Blankenstein

## Data Availability

No datasets were generated or analysed during the current study.
